# Low Lattice Thermal Conductivity of a Two-Dimensional Phosphorene Oxide

**DOI:** 10.1038/s41598-019-41696-y

**Published:** 2019-03-26

**Authors:** Seungjun Lee, Seoung-Hun Kang, Young-Kyun Kwon

**Affiliations:** 1 Department of Physics and Research Institute for Basic Sciences, Kyung Hee University, Seoul, 02447 Korea; 2 Present Address: Korea Institute for Advanced Study (KIAS), Seoul, 02455 Korea

## Abstract

A fundamental understanding of the phonon transport mechanism is important for optimizing the efficiency of thermoelectric devices. In this study, we investigate the thermal transport properties of the oxidized form of phosphorene called phosphorene oxide (PO) by solving phonon Boltzmann transport equation based on first-principles density functional theory. We reveal that PO exhibits a much lower thermal conductivity (2.42–7.08 W/mK at 300 K) than its pristine counterpart as well as other two-dimensional materials. To comprehend the physical origin of such low thermal conductivity, we scrutinize the contribution of each phonon branch to the thermal conductivity by evaluating various mode-dependent quantities including Grüneisen parameters, anharmonic three-phonon scattering rate, and phase space of three-phonon scattering processes. Our results show that its flexible puckered structure of PO leads to smaller sound velocities; its broken-mirror symmetry allows more ZA phonon scattering; and the relatively-free vibration of dangling oxygen atoms in PO gives rise to additional scattering resulting in further reduction in the phonon lifetime. These results can be verified by the fact that PO has larger phase space for three-phonon processes than phosphorene. Furthermore we show that the thermal conductivity of PO can be optimized by controlling its size or its phonon mean free path, indicating that PO can be a promising candidate for low-dimensional thermoelectric devices.

## Introduction

The thermoelectric effect is one of the promising next-generation energy harvesting technologies and has been studied extensively in recent decades^1,2^. The efficiency of a thermoelectric device can be expressed using a dimensionless quantity, *ZT* defined as

$$ZT=\frac{{S}^{2}\sigma T}{\lambda +\kappa },$$

where *S*, *σ*, *T*, *λ*, and *κ* represent the Seebeck coefficient, electrical conductivity, absolute temperature, electron and lattice thermal conductivities, respectively. In order to increase *ZT* value, we need to increase the power factor *S*^2^*σ*, and at the same time to decrease thermal conductivities. Seebeck coefficient *S* is usually much higher in semiconductor than in metal, since *S* is strongly related to differential total electron density of state (DOS), which is maximized near band gaps, where DOS changes abruptly. On the other hand, it is, unfortunately, impossible to adjust *σ* high, and *λ* low simultaneously to enhance the thermoelectricity, because they are coupled to each other and governed by the Wiedemann-Franz law. In semiconductors, the phononic contribution of the thermal conductivity (*κ*) is much larger than its electronic contribution (*λ*). Moreover, *κ* is partially decoupled from *σ*, and thus its reduction is a useful strategy for high-performance thermoelectric materials. Recently, a few studies proposed that introduction of particular defect structures increases *σ*/(*λ* + *κ*) since the thermal conductivity decreases much more than its electrical counterpart, although the defects usually reduce both^3,4^. Another interesting research showed that making low-dimensional structures enhances thermoelectric power. In the low-dimensional materials, *S* can be further increased due to its unique properties, such as sudden changes in the DOS^5^, and quantum confinement effects^6,7^. Moreover, such structural redesign may affect thermal conductivity. *κ* can be lowered more while keeping *σ* by forming superlattice or nanocrystal structures^8,9^, because the mean free path of phonon is longer than that of electron. To realize state-of-the-art thermoelectric devices, therefore, one should design a quasi low-dimensional or superlattice structure with a semiconductor, which has a low lattice thermal conductivity. It is, for example, known that the best thermoelectric materials are Bi_2_ Sb_3_/Sb_2_ Te_3_ superlattice^10^ and layered SnSe structure^11^, which exhibit the highest *ZT* values.

Phosphorene, which has recently attracted attentions in various fields related to materials research^12,13^, would fit in a category satisfying conditions mentioned above. Due to its puckered structure representing strong anisotropy, its mechanical, electrical and thermal properties shows remarkably different directional behavior, especially along the armchair and zigzag directions^14–19^. There have been several reports showing that phosphorene has a high *ZT* value along the armchair direction^20,21^.

However, the poor stability of phosphorene under ambient air conditions has been a crucial problem for its various applications including thermoelectric devices. Earlier studies reported that phosphorene can be easily oxidized in air because of the lone-pair electrons in each phosphorus atom^22–25^. Although there were a few proposals to prevent phosphorene from being oxidized^26^, they are not easily applicable, and thus it is still difficult to use phosphorene as a thermoelectric device. Another way to overcome the poor air stability of phosphorene is to use the oxidized structure itself as a device. It was reported that a particular structure of phosphorene oxide (PO) is a semiconductor with a direct band gap of 0.6~0.88 eV^23–25^, which is slightly smaller than that of its pristine counterpart, phosphorene. It was also reported that PO has a relatively small electron effective mass of 0.18 *m*_*e*_^24^, which is similar to that of phosphorene along the armchair direction and much smaller than along the zigzag direction. In addition, phosphorene oxide exhibits some interesting physical properties such as non-symmorphic phase protected band structure^25^.

Moreover, PO satisfies the conditions mentioned above for good thermoelectric materials. In this paper, we report our investigation on the thermal transport properties of the oxidized structure of phosphorene by solving the Boltzmann transport equation based on first-principles density functional theory. PO shows significantly lower lattice thermal conductivity than phosphorene. Furthermore, we present our analysis of why such low lattice thermal conductivity was observed in PO. Our analysis includes the evaluation of the phonon relaxation time, Grüneisen parameters, and phase space of the three-phonon processes, all of which enable us to understand the fundamental origin of the low thermal conductivity of PO.

## Results and Discussion

**Figure 1:**
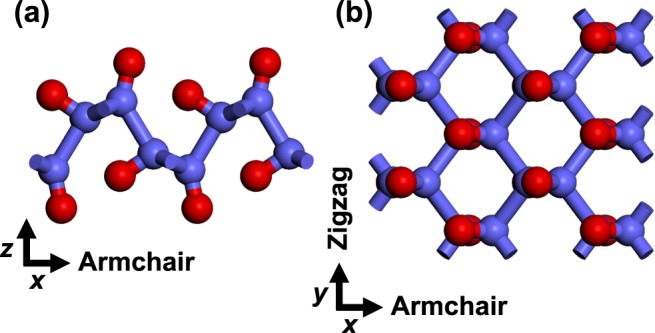
Equilibrium structure of PO shown in side (**a**) and top (**b**) views. Phosphorus and oxygen atoms are depicted by blue and red spheres.

In an earlier study^25^, we found the equilibrium structure of PO, as shown in Fig. 1. Similar to phosphorene, PO has a puckered configuration, composed of four phosphorus and four oxygen atoms in a primitive unit cell. Every P atom is connected to three other P atoms and one O atom, whereas every O atom to one P atom and has three lone pairs of electrons. The optimized lattice constants along the armchair and zigzag directions were calculated to be 5.12 and 3.66 Å, respectively, in good agreement with previous studies of PO^23,24^. We expanded this equilibrium structure to 4 × 4 × 1 super cell to investigate the thermal properties.

**Figure 2:**
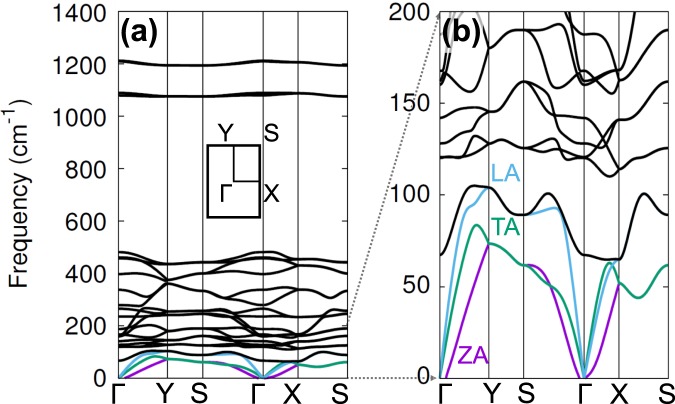
Phonon dispersion of PO in (**a**) the whole frequency range and in (**b**) the low frequency region (*ω* ≤ 200 cm^−1^). The three acoustic branches (LA: longitudinal, TA: transverse, ZA: flexural modes) are represented respectively by sky blue, green, and purple colors. The BZ of PO and the special path and points are shown in the inset in (**a**).

**Figure 3:**
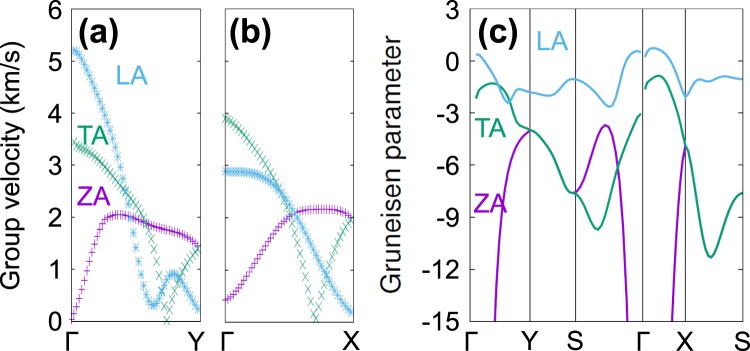
Absolute values of phonon group velocities of PO for the three acoustic branches along (**a**) the Γ-Y and (**b**) the Γ-X directions. (**c**) Grüneisen parameters calculated for the three acoustic modes in the first BZ. These calculated values for three modes LA, TA, and ZA are depicted by sky blue, green and purple colors, respectively. To compensate for the imaginary frequencies found in the ZA mode, which should exhibit the quadratic behavior near Γ, its group velocity along the Γ-Y direction was fitted using a polynomial function.

Figure 2 shows our calculated phonon dispersion of PO plotted along the special lines shown in its inset in (a). We focused more on three acoustic branches (LA: longitudinal, TA: transverse, ZA: flexural modes), which are main contribution to the thermal conductivity. As shown in Fig. 2(b), which is zoomed in below *ω* ≤ 200 from (a), the acoustic modes exhibit anisotropic dispersion along the Γ-Y (zigzag) and Γ-X (armchair) directions. To clearly describe such an anisotropic behaviors in the dispersion, we computed the group velocity *v*_g_ of the three acoustic branches along these two directions. As shown in Fig. 3(a,b), the sound velocities of LA and TA modes are in the range of 2.9–5.2 km/s, which are slightly smaller than those of other 2D materials such as silicene (5.4–8.8 km/s)^27^, MoS_2_ (4.2–6.8 km/s)^28^, graphene (3.7–6.0 km/s)^29^, and phosphorene (4.0–7.8 km/s)^16^. We found that *v*_g_ of the LA phonon mode along the zigzag direction is larger than that along the armchair direction, as we easily understand since the LA mode depends strongly on the elastic modulus of the material. Due to the puckered structure shown in Fig. 1(a), PO can be more easily stretched or compressed along the armchair direction compared to along their zigzag counterpart, similar to phosphorene. On the other hand, the group velocities of the TA mode do not show strong directional dependence, and so do those of the ZA mode exhibiting the quadratic behaviors as in planar graphene. Therefore, the LA mode should be responsible for the anisotropicity in thermal transport property of PO.

In a carrier transport phenomenon in a crystal solid, not only the group velocity but the carrier lifetime or scattering rate also play a crucial role in determining its transport properties. The evaluation of the carrier lifetime requires anharmonic phonon-phonon scattering or Umklapp process evolving three phonons, since the momentum-conserved phonon scattering process described by only two phonons is not relevant to the phonon lifetime.

We noticed that the Grüneisen parameter (GP), which is usually considered for the thermal expansion behaviors, gives us a useful information on the anharmonic phonon scattering rate. The mode-dependent GP is defined as

1 $${\gamma }_{j}({\bf{q}})=-\,\frac{\partial \,\mathrm{log}\,{\omega }_{j}({\bf{q}})}{\partial \,\mathrm{log}\,V},$$

Figure 3(c) shows the GP values evaluated for the three acoustic phonon branches. The ZA-mode GP *γ*_ZA_ becomes very large in the long wavelength region near the Γ point as in other 2D materials^30^ as described by membrane effect^31^. It is also clear that the GPs are largely anisotropic and discontinuous at the Γ point, reflecting its structural features similar to the sound velocity. It was derived that the mode-dependent anharmonic phonon lifetime is inversely proportional to the mode-dependent GP squared or ${\tau }_{{\rm{U}}}(j,{\bf{q}})\propto \frac{1}{|{\gamma }_{j}({\bf{q}}{)|}^{2}}$^32–35^. Thus LA mode of PO, which has smaller GP values than the TA or ZA, may exhibit longer phonon lifetime than the other acoustic modes. Therefore, the LA mode plays an important role in PO’s thermal transport behavior as we will later describe in detail.

**Figure 4:**
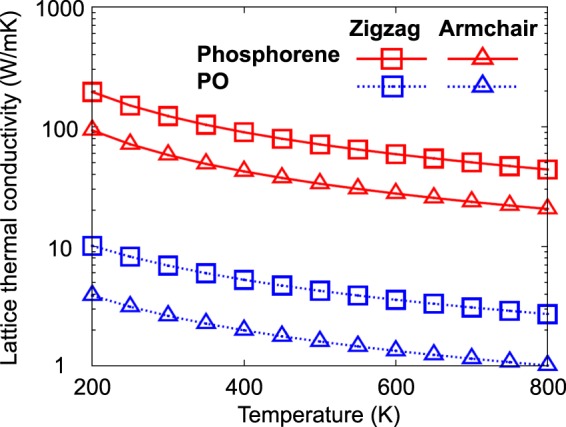
Calculated lattice thermal conductivities of phosphorene (red symbols) and PO (blue symbols) along zigzag (squares) and armchair (triangles) directions as a function of temperature ranging from 200 K to 800 K. The data points in each category were well fitted with an inverse function *a*/*T* with a single parameter *a*, plotted with the solid or dashed line.

To investigate the temperature dependence of the lattice thermal conductivity *κ*_PO_(*T*) of PO, we solved phonon Boltzmann equation using the iterative approach mentioned in the Method section. We also applied the same method to evaluate the lattice thermal conductivity *κ*_P_(*T*) of phosphorene for comparison. Figure 4 shows ${\kappa }_{{\rm{PO}}}^{{\rm{A}}}(T)$, ${\kappa }_{{\rm{PO}}}^{{\rm{Z}}}(T)$, ${\kappa }_{{\rm{P}}}^{{\rm{A}}}(T)$, and ${\kappa }_{{\rm{P}}}^{{\rm{Z}}}(T)$, where A and Z indicate the armchair and zigzag directions along which the thermal conductivities were calculated. These calculated values were almost perfectly fitted with an inverse function *a*/*T* with a single parameter *a*, which is expected from the three-phonon anharmonic process^36^. Our predicted room-temperature thermal conductivity values of phosphorene, which are 146 W/mK and 65 W/mK along the zigzag and armchair directions, respectively, agree reasonably well with previously reported values ranging from 110 to 153 W/mK and from 33 to 64 W/mK along the respective directions^18,19,37^. Both phosphorene and PO exhibit that their lattice thermal conductivities along their armchair directions are approximately 2.5 times smaller than those along the zigzag directions, owing to the structural anisotropy mentioned above. Although PO is structurally similar to phosphorene, we observed that the thermal conductivity values of PO are much lower than those of phosphorene over the whole temperature range regardless of the transport directions. For example, at room temperature (*T* = 300 K), we found ${\kappa }_{{\rm{PO}}}^{{\rm{A}}}$ to be 2.42 W/mK, which is lower than the values not only of phosphorene (${\kappa }_{{\rm{P}}}^{{\rm{A}}}=65$ W/mK), but also of other 2D materials such as silicene (26 W/mK)^38^, MoS_2_ (23.2~34.5 W/mK)^39–41^, stanene (11.6 W/mK)^42^, graphene (3000~6000 W/mK)^43,44^, and hexagonal boron nitride (hBN) (350~600 W/mK)^44,45^. Low thermal conductivity is one of sufficient conditions for high-performance thermoelectric materials. In this sense, PO can be a new candidate for 2D thermoelectric materials.

To explore what causes such a low lattice thermal conductivity in PO, we first estimated the contributions of various phonon modes to the *κ*_PO_ and summarized in Table 1. It was revealed that the ZA phonon mode has a significantly long lifetime in a completely flat 2D material because its mirror symmetry limits the phase space for phonon-phonon scattering of the ZA phonon mode^46^. For example, in graphene and hBN, whose room-temperature thermal conductivities have been reported to be a few thousands^43,46^ and a few hundreds^44,45^ in W/mK, their ZA modes contribute to their thermal conductivities by about 75%^44,46^. We found, on the other hand, that the contribution of the ZA mode in PO becomes less significant to be only 15~17%, whereas the contribution from the other modes including the LA and TA modes becomes dominant. This is because PO is not a perfect 2D structure, but a puckered one, allowing more phonon-phonon scattering of the ZA mode. Thus, puckered structures would be advantageous for a low-dimensional thermoelectric application.

**Table 1:** Contribution of phonon branches in % to the lattice thermal conductivities of PO at 300 K along the zigzag and armchair directions, ${\kappa }_{{\rm{PO}}}^{{\rm{A}}}$ and ${\kappa }_{{\rm{PO}}}^{{\rm{Z}}}$.

	Contribution of phonon branches in % to	
	${{\boldsymbol{\kappa }}}_{{\bf{PO}}}^{{\bf{A}}}$	${{\boldsymbol{\kappa }}}_{{\bf{PO}}}^{{\bf{Z}}}$
LA	42	27
TA	14	30
ZA	15	17
Others	29	26

**Figure 5:**
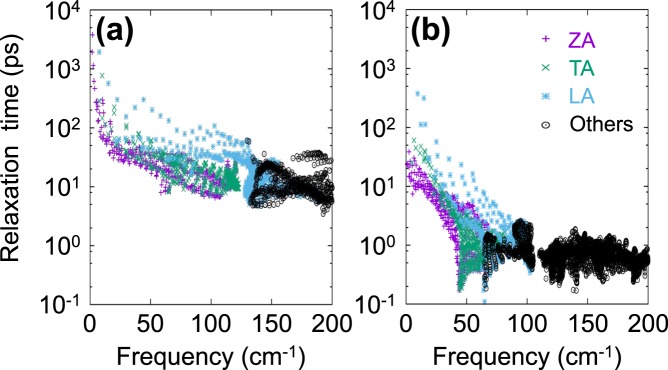
The mode-dependent anharmonic phonon relaxation time of (**a**) phosphorene and (**b**) PO for three acoustic modes, ZA (purple), TA (green), LA (blue), and all the other modes (black). The relaxation time is given in a logarithmic scale.

To further analyze the low lattice thermal conductivity of PO, we evaluated the relaxation times of phosphorene and PO as a function of frequency. In general, there are various sources giving rise to phonon scattering, for example, anharmonic phonon-phonon or Umklapp (U) scattering, phonon-electron scattering, impurity effect, boundary effect, isotope effect, and so on. Among these scattering sources, we took only the phonon-phonon scattering into account, since the other sources were much smaller in PO. Therefore, we simply replaced the total scattering rate 1/*τ* with the Umklapp scattering rate 1/*τ*_U_. Figure 5 shows our calculated mode-dependent anharmonic phonon relaxation time of phosphorene and PO for three acoustic modes (ZA, TA, and LA) and the other higher modes. The phonon lifetime of PO is more than one-tenth smaller than that of phosphorene as shown in Fig. 5. We assigned this reduction to two effects of the oxygen atoms in PO. One is the trivial P-O composite effect, and the other is due to the flexibility of oxygen atoms. Thus, oxidation occuring spontaneously on the phosphorene surface, becomes an additional advantage leading to low thermal conductivity. Each oxygen atom, which is connected only to one phosphrous atoms, is a kind of a dangling atom, whereas each phosphorous atom has four tetrahedral bonds with neighboring atoms. Thus, oxygen atoms may participate not only into their optical P − O vibration, but also vibrate along the in-plane directions together with phosphorous atoms contributing to the acoustic modes. This contribution may be responsible for the acoustic phonon softening leading to the reduction in the thermal conductivity of PO. To verify the effect of existence of such dangling atoms on the acoustic phonon modes, we devised a model structure mimicking the PO system. See Supplementary Information for our model calculation.

To discuss the physical phenomena of phonon scattering in more detail, we analyzed the allowed phase space *P*_3_ for the anharmonic three-phonon scattering process introduced by Lindsay and Broido^47^. The three-phonon scattering is allowed only when the energy and momentum conservation conditions are satisfied. Thus, the phonon scattering rate of each phonon state is proportional to the number of available final states. In other words, the phonon lifetime of each mode *ω*_*j*_(**q**) is inversely correlated with the mode-dependent phase space *P*_3_(*ω*_*j*_(**q**)) defined by^48^

2 $${P}_{3}({\omega }_{j}({\bf{q}}))=\frac{1}{{{\rm{\Omega }}}_{{\rm{BZ}}}}[\frac{2}{3}{D}_{j}^{(+)}({\bf{q}})+\frac{1}{3}{D}_{j}^{(-)}({\bf{q}})],$$

where Ω_BZ_ is the volume of BZ; ${D}_{j}^{(\pm )}({\bf{q}})$ are two-phonon densities of states^49^ for absorption (+) and emission (−) processes. Difference of a factor two between two coefficients was introduced to avoid double counting. ${D}_{j}^{(\pm )}({\bf{q}})$ can be evaluated by

3 $${D}_{j}^{(\pm )}({\bf{q}})=\sum _{j^{\prime} ,j^{\prime\prime} }\,\int \,d{\bf{q}}{\boldsymbol{^{\prime} }}\delta \,[{\omega }_{j}({\bf{q}})\pm {\omega }_{j^{\prime} }({\bf{q}}{\boldsymbol{^{\prime} }})-{\omega }_{{j}^{^{\prime\prime} }}({\bf{q}}\pm {\bf{q}}{\boldsymbol{^{\prime} }}-{\bf{G}})].$$

**Figure 6:**
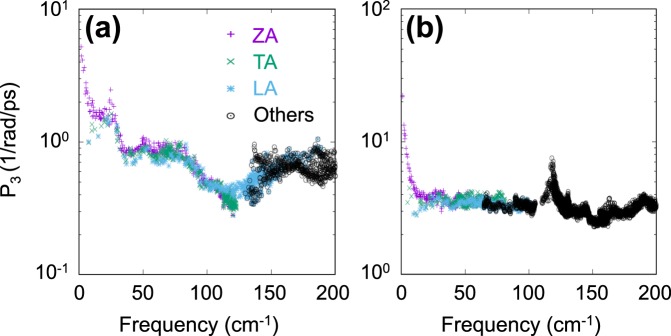
Allowed mode-dependent phase spaces *P*_3_(*ω*_*j*_(**q**)) of three-phonon scattering processes evaluated in (**a**) phosphorene and (**b**) PO for three acoustic modes ZA (purple), TA (green), LA (blue) and all the other modes (black). *P*_3_ values are given in a logarithmic scale.

Here the momentum conservation was already imposed to replace **q″** with **q ± q′** − **G**, where **G** vectors are the reciprocal lattice vectors to describe Umklapp processes. Figure 6 shows *P*_3_ values as a function of frequency *ω*_*j*_ evaluated over the Brillouin zone. It is clear that PO has a much larger *P*_3_ value than phosphorene, indicating that more scattering processes occur in PO than in phosphorene. This explains why PO has a much shorter relaxation time than phosphorene.

**Figure 7:**
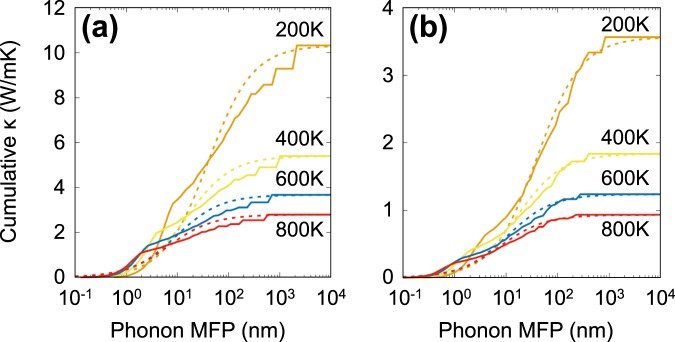
Cumulative lattice thermal conductivities (solid lines) of PO along (**a**) zigzag and (**b**) armchair directions with respect to the phonon mean free path (MFP) at various temperatures. These conductivities were fitted by the function defined in the text, plotted with dotted lines.

To design high-performance thermoelectric devices, most important strategy is to reduce the thermal conductivity while remaining its electronic counterpart. Usually the lattice thermal conductivity of a system can be decreased by reducing the size of the system, since the size reduction results in shortening of its phonon MFP. On the other hand, electron MFP is much smaller than that of phonon especially in semiconductors. For example, the electron MFP of phosphorene is known to be around only a few nm at 300 K^20^. Therefore, optimization of grain size in nanocrystalline structures or nanowire approach can be an effective way to enhance the thermoelectric figure of merit. To explore the size dependence of thermal conductivity of PO, we evaluated the cumulative thermal conductivity *κ*(*l*) by summing up all the contributions from phonon modes with MFP smaller than *l*. Figure 7 shows the computed *κ*(*l*) as a function of phonon MFP along both zigzag and armchair directions at various temperatures. As shown in the figure, *κ*(*l*) curves resemble a logistic function when MFP is given in a logarithmic scale, and thus we fit our calculated data to a single parametric function given as^50^

4 $$\kappa (l)=\frac{\kappa }{1+{l}_{0}/l},$$

where *l*_0_ is the fitting parameter determining the characteristic value of the MFP, yielding *κ*(*l* = *l*_0_) = *κ*/2. The *l*_0_ values are fitted to be 40.26 (39.98), 15.30 (16.46), 9.44 (10.48), and 6.71 (7.64) nm at 200, 400, 600, and 800 K, respectively, for the zigzag (armchair) direction.

## Summary and Conclusions

We presented our investigation on the thermal transport properties of phosphorene oxide (PO) as well as phosphorene using the first-principles calculations combining with the semiclassical Boltzmann transport theory. We found that the thermal conductivity of PO is much lower than those of other two-dimensional materials including phosphorene, revealing that oxidation is responsible for the reduction in the thermal conductivity. The thermal conductivity of PO depends strongly on its transport directions, and were calculated to be 2.42 and 7.08 W/mK along the armchair and the zigzag directions at 300 K. Similar to phosphorene, PO is structurally characterized by the flexible puckered structure, which leads to lower-frequency acoustic phonon modes and smaller sound velocities than genuine 2D flat materials. In addition, PO possesses no mirror symmetry allowing more ZA phonon scattering. Furthermore, we identified that nearly-free vibration of dangling oxygen atoms gives rise to additional scattering resulting in further reduction in the phonon lifetime. Spontaneous oxidation of phosphorene greatly reduces its thermal conductivity, which can be additionally optimized by controlling the size, and thus PO can be a promising candidate for use in low-dimensional thermoelectric devices.

## Methods

To investigate the thermal property of PO, we performed first-principles calculations based on density functional theory^51^ as implemented in the Vienna *ab initio* simulation package (VASP)^52,53^. We employed the projector augmented wave potentials^54,55^ to describe the valence electrons, and treated exchange-correlation functional within the generalized gradient approximation of Perdew-Burke-Ernzerhof ^56^. The plane-wave kinetic energy cutoff was selected to be 500 eV, and *c* = 20 Å was chosen for the lattice constant of the direction perpendicular to the plane to minimize the interlayer interaction. The Brillouin zone (BZ) of each structure was sampled using a 11 × 11 × 1 *k*-point grid for the primitive unit cells of phosphorene and PO.

To precisely evaluate the temperature dependence of the lattice thermal conductivity *κ*_PO_(*T*) of PO, we solved the phonon Boltzmann transport equation using an iterative approach proposed by Omini *et al*.^57^, which implemented in the ShengBTE code^50^. The thermal conductivity tensor *κ*^*αβ*^, where *α* and *β* denote *x*, *y* or *z*, can be obtained from

5 $${\kappa }^{\alpha \beta }=\frac{1}{{k}_{B}{T}^{2}{\rm{\Omega }}N}\sum _{j,{\bf{q}}}\,{f}_{0}({f}_{0}+\mathrm{1)(}\hslash {\omega }_{j,{\bf{q}}}{)}^{2}{v}_{j,{\bf{q}}}^{\alpha }{L}_{j,{\bf{q}}}^{\beta }$$

where *k*_*B*_ is the Boltzmann constant, Ω the unit cell volume, and *N* the Γ-centered **q**-point grids. In the summand, *f*_0_ is the Bose-Einstein distribution function; and *ω*_*j*,**q**_ and **v**_*j*,**q**_ are respectively the phonon frequency and group velocity of the phonon mode with the branch index *j* and wavevector **q**. **L**_*j*,**q**_ was introduced to compensate the phonon distribution deviated from *f*_0_ in the presence of the temperature gradient. This quantity with a dimension of length can be expressed as

6 $${{\bf{L}}}_{j,{\bf{q}}}={\tau }_{j,{\bf{q}}}^{0}({{\bf{v}}}_{j,{\bf{q}}}+{{\bf{u}}}_{j,{\bf{q}}}),$$

where ${\tau }_{j,{\bf{q}}}^{0}$ is the single-mode relaxation time (SMRT) estimated from the imaginary part of self energy obtained by many body perturbation theory including the anharmonic three phonon scattering. Here, **u**_*j*,**q**_ is the correction to the SMRT approach due to the deviation of the phonon population at the specific mode with *j* and **q** from the Bose-Einstein statistics. Since it is nonlinearly coupled with **L**_*j*,**q**_, we solved Eq. (6) iteratively to evaluate **u**_*j*,**q**_ and thus **L**_*j*,**q**_. For more complete expressions of ${\tau }_{j,{\bf{q}}}^{0}$, **L**_*j*,**q**_, and **u**_*j*,**q**_, and their evaluation approach, see the reference [ShengBTE_2014]. The phonon dispersion relations of phosphorene and PO were calculated by applying the finite displacement method^58^ to their respective 6 × 6 × 1 supercells. We also computed their third-order interatomic force constants and phonon relaxation times to evaluate their correpsonding lattice thermal conductivities^50,59^ using 4 × 4 × 1 supercell. We took up to the fourth nearest neighboring interaction and the corresponding BZ was sampled by a 2 × 2 × 1 *k*-point grid. Note that the cross-section area should be determined in order to evaluate the thermal conductivity. As usually done in two dimensional cases, the thicknesses of phosphorene and PO were set to 5.5 and 8.0 Å,respectively, approximately corresponding to interlayer distances in their bulk configurations.

## Supplementary information

Low Lattice Thermal Conductivity of a Two-Dimensional Phosphorene Oxide

## Data Availability

The datasets generated during and/or analysed during the current study are available from the corresponding author on reasonable request.
