# Supplementary material for: Low Lattice Thermal Conductivity of a Two-Dimensional Phosphorene Oxide
Source: Sci Rep. 2019 Mar 26;9:5149. doi: 10.1038/s41598-019-41696-y (PMC6435745; doi:10.1038/s41598-019-41696-y)
Supplement: Supplementary file 1 — Low Lattice Thermal Conductivity of a Two-Dimensional Phosphorene Oxide [file 41598_2019_41696_MOESM1_ESM.pdf]

Supplementary Information

Low Lattice Thermal Conductivity of a Two-Dimensional Phosphorene Oxide

Seungjun Lee,[1] Seoung-Hun Kang,[1,*] and Young-Kyun Kwon[1,†]

[1]*Department of Physics and Research Institute for Basic Sciences, Kyung Hee University, Seoul, 02447, Korea*

(Dated: February 24, 2019)

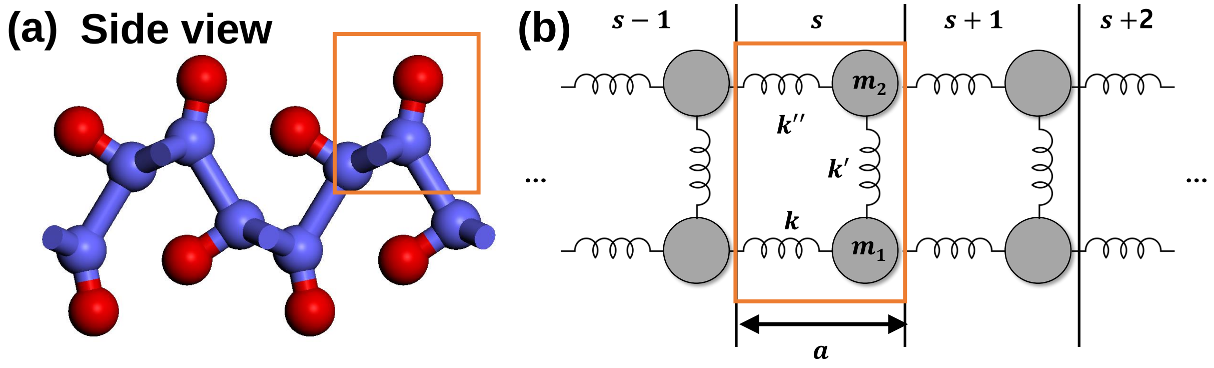

FIG. S1. (a) Side view of phosphorene oxide (PO) and (b) a simple quasi one-dimensional model structure corresponding to PO. Masses m_1 and m_2 represent phosphorous and oxygen atoms, respectively, and the spring constants k, k' and k'' do the P−P, P−O, and O−O bonds, respectively. In the limit of $k'' \to 0$, m_2 may be mapped into the "flexible" oxygen atom in PO. The box depicts the s-th unit cell with the lattice constant a of the mode structure. To simplify the model calculation, we only allowed m_1 and m_2 to move horizontally.

To investigate the role of the "flexible" oxygen atom in PO, we devised the model structure composed of two masses m_1 and m_2 connected with three different types of springs k, k', k'' as shown in Fig. S1. With the generalized coordinates u_s and v_s assigned to the displacements of the s-th m_1 and m_2, the equation of motion is given by

$$m_1 \frac{d^2 u_s}{dt^2} = k(u_{s+1} + u_{s-1} - 2u_s) - k'(u_s - v_s) \tag{S1}$$

$$m_2 \frac{d^2 v_s}{dt^2} = k''(v_{s+1} + v_{s-1} - 2v_s) + k'(u_s - v_s). \tag{S2}$$

We look for a traveling wave solution with different amplitudes u and v,

$$u_s = u e^{isqa} e^{-i\omega t}, \qquad v_s = v e^{isqa} e^{-i\omega t}.$$

Thus, Eqs. (S1) and (S2) become

$$-m_1 \omega^2 u = k(e^{iqa} + e^{-iqa} - 2)u - k'(u - v)$$

$$-m_2 \omega^2 v = k''(e^{iqa} + e^{-iqa} - 2)u + k'(u - v).$$

These coupled equations can be solved by setting the determinant to be zero, or

$$m_1 m_2 \omega^4 + \omega^2 \left\{-4(m_2 k + m_1 k'') \sin^2\left(\frac{qa}{2}\right) - k'(m_1 + m_2)\right\} + 16kk'' \sin^4\left(\frac{qa}{2}\right) + 4k'(k + k'') \sin^2\left(\frac{qa}{2}\right) = 0,$$

and thus the two solutions are

$$\omega_\pm^2 = 2\left(\frac{k}{m_1} + \frac{k''}{m_2}\right) \sin^2\left(\frac{qa}{2}\right) + \frac{k'}{2}\left(\frac{1}{m_1} + \frac{1}{m_2}\right) \pm \sqrt{4\left(\frac{k}{m_1} - \frac{k''}{m_2}\right)^2 \sin^4\left(\frac{qa}{2}\right) + \frac{k'^2}{4}\left(\frac{1}{m_1} + \frac{1}{m_2}\right)^2 + 2k' \sin^2\left(\frac{qa}{2}\right)\left(\frac{k}{m_1^2} + \frac{k''}{m_2^2} - \frac{k + k''}{m_1 m_2}\right)} \tag{S3}$$

These solution can be confirmed in a special condition of $m_1 = m_2 = m$ to be

$$\omega_\pm^2 = \frac{1}{m}\left[2(k + k'') \sin^2\left(\frac{qa}{2}\right) + k' \pm \sqrt{4(k - k'')^2 \sin^4\left(\frac{qa}{2}\right) + k'^2}\right],$$

and further to be, when $k'' = k$,

$$\omega_\pm^2 = \frac{1}{m}\left[4k \sin^2\left(\frac{qa}{2}\right) + k' \pm k'\right].$$

The lower solution ω_- simply corresponds to the acoustic phonon branch of the monatomic chain system.

To understand role of the flexible oxygen atom corresponding to m_2 and k'', we plotted the phonon dispersion relations of our model system with various parameters shown in Fig. S2. As clearly seen in the figure, the smaller k'', the lower the acoustic phonon frequency. Thus, the flexibility of oxygen atoms in PO may lead to the softening of acoustic phonon modes resulting in the reduction in thermal conductivity.

---

* Current address: Korea Institute for Advanced Study (KIAS), Seoul 02455, Korea

† Corresponding author. E-mail: ykkwon@khu.ac.kr

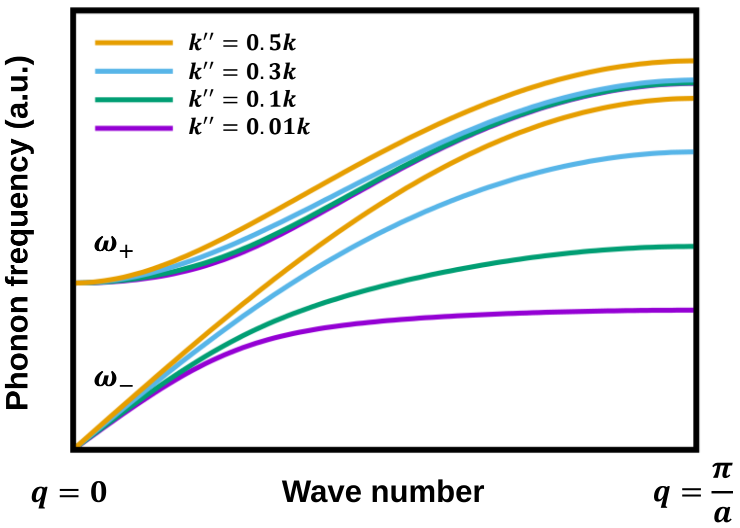

FIG. S2. Calculated phonon dispersion relations of the model system shown in Fig. S1. $\omega_\pm$ given in Eq. S3 represent the optical (+) and acoustic (−) branches, which were plotted with $m_2 = 0.5m_1$, $k' = 0.3k$, while changing k'' from $0.01k$ to $0.5k$.
